# Neuroendocrine Neoplasia: From Pathophysiology to Novel Therapeutic Approaches

**DOI:** 10.3390/biomedicines12040801

**Published:** 2024-04-04

**Authors:** Mara Carsote, Claudiu Nistor

**Affiliations:** 1 Department of Endocrinology, “Carol Davila” University of Medicine and Pharmacy, 020021 Bucharest, Romania; 2 Department of Clinical Endocrinology V, “C.I. Parhon” National Institute of Endocrinology, 011863 Bucharest, Romania; 3 Department 4-Cardio-Thoracic Pathology, Thoracic Surgery II Discipline, “Carol Davila” University of Medicine and Pharmacy, 050474 Bucharest, Romania; claudiu.nistor@umfcd.ro; 4 Thoracic Surgery Department, “Dr. Carol Davila” Central Military University Emergency Hospital, 010242 Bucharest, Romania

## 1. Introduction

Neuroendocrine neoplasia (NEN) represents a sensational field of modern medicine; immense progress in emerging biochemical, molecular, endocrine, immunohistochemical, and serum tumour markers of disease, respectively, which are part of early diagnosis, genetic testing, and multidisciplinary approaches. The management of NENs combines precision medicine with updated guidelines and protocols (sometimes provided by joint associations of practitioners with distinct backgrounds) [1,2,3,4].

The domain of NENs involves both adult and (rarely) paediatric patients; heterogeneous NENs with hereditary components or sporadic types of them, as well as NENs with different prevalence ratios from various studies, are more frequently being identified as gastro-entero-pancreatic (GEP) and lung NENs [5]. Despite NENs remaining a rather uncommon finding in the general population (currently the incidence rate is 6 per 100,000 people and the prevalence is 35 per 100,000 people), an increasing incidence of 6.4 times has been reported over the last 40 years [6,7,8].

We aimed to integrate the data provided by the studies published in this Special Issue amid recent insights into the specific framework of NENs.

## 2. Prognostic Markers and Multimodal Management of NENs

The most important prognostic markers are confined to a well-known panel that includes tumour diameter, grading (based on pathological reports and immunohistochemical analysis using the GRADE system, which is continuously updated according to World Health Organization classifications), and somatostatin receptor spectrum. Analysis of this panel of prognostic markers should be carried out using the precise configuration of one NEN in terms of its hormonal profile, localisation, and access to complete removal, etc., through a multidisciplinary team decision [1,9,10,11].

For instance, in GEP NENs, gastric tumours represent rare forms originating from stomach neuroendocrine cells, which have lately been more often identified due to frequent routine investigations such as oesophagus-gastro-duodenoscopies [1,12]. In addition to typical grades of low, intermediate, and high, they are classified as type 1 and 2 (with gastrin overproduction in relation to atrophic gastritis and with a multiple endocrine neoplasia type 1 syndrome/Zollinger–Ellison syndrome due to a gastrinoma in type 2) as well as type 3 and 4 (sporadic forms with normal gastrin). The worst prognosis is associated with the non-gastrin-derivate types [13,14].

Additionally, in gastric NENs, particular aspects such as the depth of wall infiltration, as well as the optimized (and modified) endoscopic resection versus traditional surgery (which remains the cornerstone of individualized management unless endoscopic resection cannot be safely provided) should be taken into consideration when assessing the overall outcome [2,15,16]. Also, the non-surgical approach for type 1 small gastric NENs under endoscopic surveillance has been confirmed to provide a good prognosis [17].

The presence of chronic atrophic gastritis is pivotal in gastric NEN prognosis, whereas types 1 and 2 generally have a better prognosis than type 3 (which usually constitutes advanced disease at first presentation) [1,18,19,20]. For example, Sheikh-Ahmad et al. [21] conducted a single-centric study on patients with type 1 gastric NENs who were followed up for 41 months (between 11 and 288 months). Tumour recurrence was found in 48% of the cohort after a median follow-up of 35 months. Gastrin levels were higher in a statistically significant manner in subjects with recurrence when compared to those who did not experience recurrent disease [21]. Notably, another recent multi-centric study on well-differentiated gastric NENs (N = 94, median follow-up of 49 months) showed a recurrence rate of 14%; the most important parameters that served as indexes of recurrence comprised the pre-therapy level of gastrin and the choice of therapy. In this cohort, the lowest recurrence rates were found in subjects who were treated with somatostatin analogues (SSAs) (5%) and those who underwent an endoscopic submucosal dissection (10%) [22].

Pancreatic NENs, which are classified in well-differentiated neuroendocrine tumours and poorly differentiated neuroendocrine carcinomas [23], may pose an additional challenge in subjects with functional tumours that require not only a tumour growth control but also the relief of hormonal syndromes [24]. The aforementioned panel of contributors to the recurrence of GEP NENs is also applicable for this site. Osher E et al. studied the involvement of the lymph node as a prognostic factor in 95 individuals confirmed as having pancreatic NENs who were treated with surgery (N = 78 subjects were analysed with an average age of 57.4 ± 13.4 years; 64% were males). Approximately one-third of this subgroup was confirmed as having lymph node metastases. Even if grading and staging are widely accepted as the main prognostic factors, in some studies, lymph node involvement was also regarded as a prognostic marker; however, in this study, the lymph node ratio was not confirmed as being correlated with disease recurrence (an overall 5-year disease-free survival rate of 71.8% was identified for the entire cohort) [25]. Moreover, radiofrequency thermal ablation for localized pancreatic NENs was recently introduced with an as yet undetermined contribution to the overall prognosis, and this represents a prognostic factor to explore in the future [26].

## 3. The Use of Somatostatin Analogues (SSAs)

As part of the standard therapy sequence for NENs, long-acting SSAs, such as octreotide and lanreotide, are provided to subjects with positive somatostatin receptors. They represent a significant part of medical management before and after surgery, an alternative to surgery in inoperable cases, or a long-term management option for NENs that have been recently described as “resistant” (tumours that display symptom progression and metastatic spreading despite standard care treatment) [1,2,6,27].

However, in daily practice, most candidates for whom SSAs may be used as a first-line therapy are diagnosed with G1/G2 well-differentiated NENs with a better prognosis [28]. Compliance to this treatment might potentially impact the prognostic. Recently, a study on metastatic GEP NENs showed that almost half of these patients experience subcutaneous nodules due to the route of administration, while the development of these nodules might not influence the overall survival rate [29] but might potentially affect compliance to long-term medication. The administration of SSAs constitutes a prognostic marker for NENs (other than the specific spectrum of the tumour’s profile).

Sebastian-Valles et al. [30] conducted a two-centric retrospective study on individuals confirmed as having type 1 gastric NENs who had undergone therapy with SSAs for 22 months, and 88.9% of them registered a partial or complete response. The authors showed that therapy using SSAs was the sole independent contributor in preventing NEN recurrence (odds ratio of 0.054, *p* = 0.005). The levels of serum chromogranin A and gastrin did not have a statistically significant correlation with the prognosis, but in the case of gastrin assays, the synchronous use of proton pump inhibitors might provide a bias concerning its blood concentration [30]. Another recent study on type 1 gastric NENs (76% were offered endoscopic treatment, 10.5% received SSAs, and 6.6% underwent surgery) showed a recurrence rate in 41.2% of the cohort after a median follow-up of 31 months [31].

There are only a few real-world studies to compare long-acting formulas of ocreotide with those of lanreotide. One recent, large cohort (based on the national French database) included subjects treated with lanreotide (N = 2327), and it proved to be associated with a higher median therapy duration than that observed for individuals treated with octreotide (N = 2090) as well as a lower rate of drug discontinuation [32]. On the contrary, a similar study included 105 patients receiving one of the two SSAs while being diagnosed with advanced, metastatic, well-differentiated GEP NENs. The median progression-free survival rate was similar, being 12 months (octreotide LAR) and 10.8 months (lanreotide depot) [33]. Another prospective, real-world setting study on adults with locally inoperable, metastatic GEP NENs using lanreotide depot identified a 2-year progression-free survival rate of 73% and a 2-year overall survival rate of 84%, while the rate of therapy-related adverse events was observed for 19.2% of patients, with a rate of 0% being observed for serious adverse effects [34].

## 4. Peptide Receptor Radionuclide Therapy (PRRT)

As previously mentioned, PRRT represents an important part of nuclear medicine-based approaches using targetable receptor expression (for somatostatin receptor—positive NENs) [1,2,35]. It is carried out in association with medical treatment represented by SSAs, chemotherapy, targeted therapies such as tyrosine kinase inhibitors (e.g., sunitinib), and mTOR-targeted drugs (e.g., everolimus), which are all provided for NENs with various profiles of somatostatin receptors or non-functioning tumours. After applying the first-line therapy with SSAs for gastro-intestinal NENs, PRRT is a second-line option, for instance, for pancreatic NENs (and sometimes as a third option depending on the feasibility of surgery) [36].

PRRT via [^177^Lu]Lu-DOTA-TATE represents one novel option for foregut, midgut, and hindgut GEP NENs, particularly for those displaying positive somatostatin receptors which are not controlled through SSAs [37]. In addition to the medical profile, two major practical points should be taken into consideration, which are namely the issue of PRRT accessibility, as many centres and countries do not provide PRRT, and the associated costs depending on the specific reimbursement protocols [38].

Generally, we identified four modern trends in approaching PRRT for NENs: **(A)** the integration of multi-layered management in order to find out which is the best combination/synergistic therapy use with PRRT; **(B)** the usefulness of PRRT for subjects diagnosed with high-grade NENs who are not traditional candidates for PRRT; **(C)** the hypothesis of NENs inducing a shift to a more aggressive tumour than before the application of PRRT; and **(D)** the rising interest in and value of alpha particle PRRT compared with beta-emitting PRRT for NEN patients [1,2,39,40,41,42,43,44].

**A.** Currently, not many clinical and experimental studies clearly pinpoint the optimal multimodal approach for integrating PRRT into other lines of therapy regarding an originating NEN. The study of Zellmer et al. [39] is noteworthy, which investigated the synergic effect of [^177^Lu]Lu-DOTA-TATE and everolimus in a mouse model regarding tumour growth upon the use of [^68^Ga]Ga-DOTA-TATE PET (positron emission tomography). This assessment was performed after one to four weeks. The authors confirmed the significant role of PRRT in inhibiting tumour growth, but they did not confirm the additional effect of everolimus. Of course, the extrapolation of these data requires further studies, including those that focus on the use of other imaging and lab techniques to assess the overall response in NENs [39]. A recent pilot study on somatostatin receptor-positive GEP NENs having a Ki67 proliferation index between 15% and 55%, studied the effects of PRRT, and the approach was offered either as a single-line therapy or as a combination treatment with capecitabine/temozolomide (with at least two consecutive cycles). The disease control rate was 60% compared to 90%; the median progression-free survival rate was higher in a statistically significant manner for the combined treatment (12 months compared to 26 months), hence suggesting that the multimodal sequence is more efficient than the single-line management [40].

Moreover, a prospective phase II study (LUMEN cohort) on 37 subjects with progressive GEP NENs included four cycles of [^177^Lu]Lu-DOTATATE. A median progression-free survival rate of 28 months was observed for them, with one-third of them achieving a partial response after the first cycle. Based on these data, the minimal tumour-absorbed dose after the first cycle of PRRT seems predictive of further outcomes, so it might represent the basis of individualized doses of PRRT [41].

In summary, the ongoing studies include a combination of anti-tumour therapies such as PRRT and chemotherapy (capecitabine and temozolomide)—particularly for pancreatic NENs or PRRT and tyrosine kinase inhibitors; the use of PRRT before NEN surgery; intra-venous use associated with the intra-arterial application of PRRT; and either different combinations of radiolabelled agents in PRRT (for example, [^177^Lu]Lu-DOTA-TATE and [^90^Y]Y-DOTA-TATE) or distinct doses applied over a lifespan [8,42].

**B.** A significant work in progress concerns studies on applying PRRT in well-differentiated higher-grade (G3) NENs that, in contrast with the well-differentiated G1-G2 NENs, still represent an open issue (it is noteworthy that, according to current classifications, this grading group is distinct from neuroendocrine carcinomas) [43].

**C.** Recently, a new working hypothesis was created stemming from real-life experience: a small subgroup of patients confirmed as having well-differentiated NENs who underwent [^177^Lu]Lu-DOTA-TATE might experience a shift into a more aggressive tumour behaviour which is similar to that of a neuroendocrine carcinoma. One recent study identified a rate of 7 out of 152 patients displaying this apparent process of dedifferentiation following the first cycle of PRRT after a median of 8.2 months. This aspect is another topic to be further explored until a definitive confirmation of this hypothesis is reached, including the identification of the risk factors for and the pathogenic contributors to this shift [44].

**D.** A highly relevant topic in the field that should be mentioned is that regarding the use of alpha- versus beta-emitting radionuclides for PRRT. Since NENs are a divergent category of tumours, beta particle-based PRRT such as [^177^Lu]Lu-DOTA-TATE is useful for a distinct group of individuals confirmed as having advanced, metastatic, and unresectable NENs. However, a subgroup of these PRRT candidates shows a lack of response to beta particles; thus, they may be more suitable for alpha particle therap (APT) such as ^225^Actinium (^225^Ac)-DOTA-TATE and ^213^Bismuth (^213^Bi)-DOTA-TOC. Due to the novelty of this PRRT sub-domain, the statistical data are still scarce. Yet, APT might overcome beta particle resistance to PRRT; however, more data are necessary with respect to haematological, renal, and liver tolerance to these radiopharmaceuticals. Currently, APT remains a second option following the mostly used ^11^In-, ^90^Y-, and ^177^Lu-labelling PRRT. Notably, APT has the advantage of a shorter range than the beta particle therapy, so a higher selective ablation is expected, as is an increased linear energy transfer as a direct contributor to neuroendocrine cell death. While no ideal PRRT strategy has been designed yet, in the years to come, APT might find its way into daily practice [45,46,47,48,49].

## 5. Conclusions

The field of NENs remains a diverse and challenging chapter of multidisciplinary medicine. The workflow of patients may be different from one centre to another, while the standard care in terms of surgery, SSAs, PRRTs, and targeted therapies remains the main focus points of the overall management of NENs. Novel approaches vary within the use of personalized medicine, which might become the new normal for typical as well as exceptional NENs with unexpected behaviour, while additional prospective studies should provide excellent evidence-based data to achieve an optimal strategy for the long-term benefit of patients.
